# Effect of the iExaminer Teaching Method on Fundus Examination Skills: A Randomized Clinical Trial

**DOI:** 10.1001/jamanetworkopen.2019.11891

**Published:** 2019-09-20

**Authors:** Kiyoshi Shikino, Shingo Suzuki, Yusuke Hirota, Makoto Kikukawa, Masatomi Ikusaka

**Affiliations:** 1 Department of General Medicine, Chiba University Hospital, Chiba, Japan; 2 Department of Medical Education, Kyushu University, Fukuoka, Japan

## Abstract

This randomized clinical trial compares the effect of the iExaminer teaching method on fundus examination skills with the traditional teaching method.

## Introduction

Studies have shown that mastery of fundus examination skills is not as high as desired among general practitioners, which implies that innovative teaching methods are needed^1,2^ because there is no way for a teacher to verify if learners have obtained a proper view of the fundus.^3,4^ Sharing a visual field between learners and their teacher would facilitate the acquisition of those skills.

The iExaminer system consists of a PanOptic ophthalmoscope, an iPhone (Apple Inc), an adapter, and an application.^5^ This system allows learners and their teacher to share the same visual perspective. Our objective was to compare the effect of the iExaminer teaching method on fundus examination skills compared with the traditional teaching method (Figure).

**Figure.**
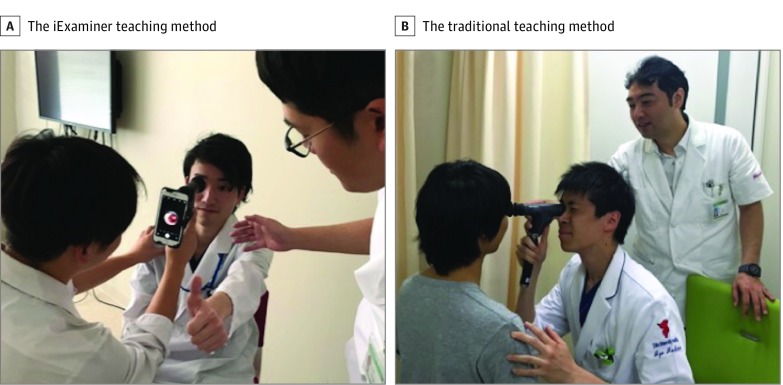
The iExaminer Teaching Method vs the Traditional Teaching Method A, Faculty taught fundus examination skills while sharing a screen with the students. B, Faculty taught fundus examination skills while students described what they saw. As guidance, faculty advised students about their grip, posture, procedure, angle, and light intensity in each group. All participants had watched instructional videos on standard use of the PanOptic ophthalmoscope and had been prepared for the interpretation of typical fundus findings 1 day before the educational session.

## Methods

A randomized clinical trial was designed to compare the effect of the iExaminer teaching method on fundus examination skills with the traditional teaching method. The trial protocol is available in Supplement 1. Participants consisted of medical students in a general medicine clinical clerkship rotation from September 2017 to July 2018. They were randomly assigned through simple randomization using Excel 2010 (Microsoft Corp) to the iExaminer method group (intervention group) or the traditional method group (control group). All instruction time was standardized to 30 minutes. Specially developed, identical training on the iExaminer method was provided to 3 instructors, who taught students using the iExaminer teaching method or the traditional teaching method. They were randomly assigned to the teaching method for each session.

The participants examined EYE Examination Simulator (Kyoto Kagaku Co) using a PanOptic ophthalmoscope before and soon after an educational session. In tests before and after training, participants were assigned 3 cases and observed 1 eye for 90 seconds. The diagnostic accuracy of ophthalmoscopic findings (ie, normal fundus, optic disc edema, pathological optic disc cupping, or not observed) and time taken to identify ophthalmoscopic findings were assessed in both tests.

Statistical analyses were performed using SPSS Statistics for Windows version 22.0 (IBM), with the level of significance set at *P* < .05. All tests were 2-tailed. Diagnostic accuracy and time were compared between the groups using a 2-way analysis of variance. Assuming an α error of .05, β error of 0.2, power of detection of 0.8, and effect size *F* of 0.25, a sample size of 128 tests was required for each group to allow for the comparison. This study was approved by the Ethics Committee of Chiba University School of Medicine. Written informed consent was obtained from all participants. This study followed the Consolidated Standards of Reporting Trials (CONSORT) reporting guideline, and the flow diagram is available in the eMethods in Supplement 2.

## Results

The total number of participants was 115. They had a median (interquartile range) age of 23 (21-31) years, and 81 (73.9%) were men. We found no statistically significant differences in demographic characteristics between the intervention and control groups. No participants had a prior clinical clerkship rotation in ophthalmology.

A 2-way analysis of variance revealed a significant effect of this intervention on diagnostic accuracy and time (Table). In the intervention group, compared with the control group, diagnostic accuracy was significantly higher (47.0% vs 30.0%; *P* = .002) and time was significantly shorter (mean [SD], 70.1 [21.9] seconds vs 76.2 [20.2] seconds; *P* = .006).

**Table.** Diagnostic Accuracy and Time Taken to Identify Ophthalmoscopic Findings

Outcome	Intervention Group (n = 59)		Control Group (n = 56)		2-Way ANOVA	
	Pretraining Test	Posttraining Test	Pretraining Test	Posttraining Test	*F*_1343_	*P* Value
Diagnostic accuracy, No. correct/total No. (mean % correct)	43/177 (24.0)	88/177 (47.0)	42/168 (25.0)	51/168 (30.0)	10.19	.002
Time taken to identify ophthalmoscopic findings, mean (SD), s	82.8 (13.1)	70.1 (21.9)	83.0 (13.6)	76.2 (20.2)	7.71	.006

Abbreviation: ANOVA, analysis of variance.

## Discussion

This study suggests that the iExaminer teaching method could be superior to the traditional teaching method for fundus examination training among medical students. The challenge for teaching the fundus examination is that teachers cannot easily determine how well the student sees the fundus.^3,4^ Students may not perform with confidence, potentially leading to the examination’s underuse.^6^ Shared visualization may explain the improved accuracy among the intervention group, but it could also be that better visualization by the student, independent of the presence of the teacher, led to this result. Other studies have successfully used covisualization techniques between learners and teachers in assessments of skill acquisition.^4^ The iExaminer teaching method may offer advantages, as demonstrated by the objective assessments in this study.

There are some potential limitations. First, this research was assessed in the simulator, not with real patients. Second, there is a possibility that the educational effect may depend on the teaching skill of faculty.

## Conclusions

In this randomized clinical trial, the iExaminer teaching method led to improved diagnostic accuracy and reduced examination time for the fundus examination. The results suggest that the iExaminer teaching method may be superior to the traditional teaching method.

## Notes

Supplement 1. **Trial Protocol**

Supplement 2. **eMethods.** Study Flow Diagram

Supplement 3. **Data Sharing Statement**
