# Supplementary material for: Effect of the iExaminer Teaching Method on Fundus Examination Skills: A Randomized Clinical Trial
Source: JAMA Netw Open. 2019 Sep 20;2(9):e1911891. doi: 10.1001/jamanetworkopen.2019.11891 (PMC6755710; doi:10.1001/jamanetworkopen.2019.11891)
Supplement: Supplement 1. — Trial Protocol [file jamanetwopen-2-e1911891-s001.pdf]

Protocol

Does iExaminer teaching method improve fundus examination skills? A randomized controlled trial

Protocol Number: 2716
Status: Approved
Initial Submit Date: 5/8/2017
Approval Period: 9/1/2017 – 8/31/2019

Principal Investigator:
Kiyoshi Shikino, MD, PhD
Department of General Medicine
Chiba University Hospital
Chiba. Japan

TABLE OF CONTENTS

Table of Contents

1. Background
2. Study objectives
3. Selection of study subjects
4. Method
5. Study period
6. Expected advantage and disadvantage of the study
7. Informed consent
8. Handling research in situations where there is an urgent and obvious crisis in the subject
9. How to deal with health hazards
10. How to protect personal information
11. Research funds and conflicts of interest
12. Possible secondary use of samples and information and provision to other research institutions
13. Research organization
14. Storage of records
15. Presentation method of research results
16. Economic burden or reward for the research subjects
17. References

Does iExaminer teaching method improve fundus examination skills? A randomized controlled trial

1. Background

All generalist physicians are required to be proficient in fundus examination for detecting prevalent diseases such as diabetic retinopathy, hypertensive retinopathy, papilledema, and retinal hemorrhage.[1,2] However, it is difficult to provide training in the necessary clinical skills for the fundus examination using the traditional teaching method[3,4] because there is no way to verify if the student has obtained a proper view of the fundus.[5,6]

We assumed that sharing of visual filed between medical students and the teacher would facilitate the acquisition of those skills. We introduced the iExaminer system to our fundus examination teaching method. The iExaminer system consists of three core components: PanOptic ophthalmoscope, iExaminer adapter, and iExaminer application. This system allows medical students and their teacher to share the same visual perspective.

2. Study objectives

The objective of our research is to investigate whether the iExaminer teaching method (using the iExaminer system) is superior to the traditional teaching method (not using the iExaminer system) for training in fundus examination.

3. Selection of study subjects

a. Selection criteria

This study's participants will consist of 115 medical students in a general medicine clinical clerkship rotation from September 2017 to July 2018. All data will be collected from those who have consented to the study. This program can be taken by all eligible students and can be taken with or without research consent.

b. Exclusion criteria

Those who have not given consent to participate in this study are excluded from the subjects.

4. Method

Procedures

This study was approved by the Ethics Committee of Chiba University School of Medicine (Chiba, Japan). A detailed explanation of the study will be given to all participants, who confirm that they fully understood the information before voluntarily giving informed consent to participate.

Study Design, Setting, and Participants

A randomized controlled trial is designed to compare the effect of the iExaminer teaching method with the traditional method for teaching the fundus examination. This study's participants will consist of 115 medical students in a general medicine clinical clerkship rotation from 2017 to 2018. The participants will be randomly assigned to either the teaching method group using the iExaminer system (intervention group) or the teaching method not using the iExaminer system (control group). As guidance, faculty advise students about their grip, posture, procedure, angle, and light intensity in each group. For the iExaminer teaching method, faculty teach fundus examination skills using the iExaminer system while sharing screens with students. On the other hand, in the traditional teaching method, faculty teach fundus examination skills while students describe what they are seeing All instruction time will be standardized to 30 minutes. Three different teachers will be randomly assigned to each session. We create the instructional design and train the faculty to minimize the educational effects.

The participants examine the fundus on the retina simulator (EYE Examination Simulator®; Kyoto Kagaku Co, Kyoto, Japan) using PanOptic ophthalmoscope before and after the educational session (pre-test and post-test). All participants will have watched instructional videos on standard use of the PanOptic ophthalmoscope and will have been prepared for the interpretation of typical fundus findings before the pre-test. In the pre-test and post-test, the students will be assigned three cases using a retina simulator. They will observe one eye for 90 seconds in each case. For each case, they will respond to a multiple-choice question asking if the case was a normal fundus, optic disc edema, pathological optic disc cupping, or not observed. The diagnostic accuracy of funduscopic findings and time taken to identify funduscopic findings will be assessed in pre-test and post-test.

Statistical Analysis

Statistical analyses will be performed using SPSS Statistics for Windows 22.0 (IBM Corp. Armonk, NY), with the level of significance set at $P < 0.05$. Diagnostic accuracy in funduscopic findings and time taken to identify funduscopic findings will be compared between the groups by using the two-way analysis of variance (ANOVA). Assuming an α error of 0.05, β error of 0.2, power of detection of 0.8, and effect size f of 0.25, a sample size of 128 tests (a total of 180 tests would be done in each group) will be required for each group to allow for comparison of the diagnostic accuracy of fundus examination.

5. Study period

a. Sub Subject intervention period 9/1/2017 – 8/31/2019
b. Subject registration period 9/1/2017 – 7/31/2019
c. Research period

6. Expected advantage and disadvantage of the study

a. Advantage
The subjects will be trained to acquire fundus skills.

b. Disadvantage
   In the control group, care should be taken so as not to cause any disadvantages by performing iExaminer teaching method after the end of the study.

7. Informed consent

This study was approved by the Ethics Committee of Chiba University School of Medicine (Chiba, Japan). A detailed explanation of the study was given to all participants, who confirmed that they fully understood the information before voluntarily giving informed consent to participate.

8. Handling research in situations where there is an urgent and obvious crisis in the subject

This research shall be conducted with the approval of the Chiba University Graduate School of Medicine Ethics Review Committee and the Chiba University Graduate School of Medicine Conflict of Interest Committee. Specifically, it will be enforced based on guidelines for clinical research, the Personal Information Protection Act, and guidelines for the proper handling of personal information in medical and nursing care-related businesses. In addition to the purpose and content of this study, the doctor in charge verbally states that the subject is free to cooperate in this study and that the subject will not suffer any disadvantages by not cooperating with the study. In addition, the questionnaires will be distributed and medical interviews will be conducted only for those who explained in writing and obtained consent.

9. How to deal with health hazards

Since this research targets information obtained within the scope of normal medical examinations, it does not perform any invasive behavior for direct research purposes. Therefore, there will be no disadvantage caused by this study.

10. How to protect personal information

Delete personally identifiable information and comply with anonymization. Although the survey form of the target person is used for data, it tries to protect personal information by anonymizing. The principal investigator is required to ensure the reliability of essential documents related to the implementation of research, etc. Documents or records etc.) shall be preserved and discarded 10 years after the publication of the research. All data is stored in a locker with a lock in the General Medical Department, Chiba University Hospital.

11. Research funds and conflicts of interest

There is no “possible conflict of interest” in the study planning, implementation, and reporting that will affect the outcome of the study and the interpretation of the results, and the conduct of the study will not harm the subject's rights or interests make sure.

12. Possible secondary use of samples and information and provision to other research institutions

None.

13. Research organization

Chiba University Hospital, Department of General Medicine		
Professor	Masatomi Ikusaka	Concept and design, Acquisition, analysis, or interpretation of data, Supervision.
Project Assistant Professor	Kiyoshi Shikino	Concept and design, Acquisition, analysis, or interpretation of data, Drafting of manuscript, Statistical analysis.
Project Assistant Professor	Shingo Suzuki	Concept and design, Acquisition, analysis, or interpretation of data, Statistical analysis, Supervision.
Project Assistant Professor	Yusuke Hirota	Concept and design, Acquisition, analysis, or interpretation of data, Drafting of manuscript Supervision.
Kyushu University, Department of Medical Education		
Makoto Kikukawa	Associated Professor	Concept and design, Acquisition, analysis, or interpretation of data, Supervision.

14. Storage of records

The principal investigator (KS) must provide the necessary documents for conducting research, etc. (a copy of the application documents, written notifications from the director of the research institute, copies of various applications and reports, consent forms, and other data necessary to guarantee the reliability of data. Documents or records etc.) shall be preserved and discarded 10 years after the publication of the research. All data is stored in a locker with a lock in the medical office of the Chiba University Hospital.

15. Presentation method of research results

The results of this study will be presented by KS at a national academic conference (Japan Primary Care Association) or an international conference (American College of Physicians Internal Medicine Meeting) after tabulation and analysis.

16. Economic burden or reward for the research subjects

There is no financial burden or reward for the subjects.

17. References

1. Day LM, Wang SX, Huang CJ. Nonmydriatic fundoscopic imaging using the pan optic iExaminer system in the pediatric emergency department. Acad Emerg Med. 2017;24(5):587-94.
2. Mottow-Lippa L. Ophthalmology in the medical school curriculum: reestablishing our value and effecting change. Ophthalmology. 2009;116(7):1235-6.
3. Mottow-Lippa L, Boker JR, Stephens F. A prospective study of the longitudinal effects of an embedded specialty curriculum on physical examination skills using an ophthalmology model. Acad Med. 2009;84(11):1622-30.
4. Wu EH, Fagan MJ, Reinert SE, Diaz JA. Self-confidence in and perceived utility of the physical examination: a comparison of medical students, residents, and faculty internists. J Gen Intern Med. 2007;22(12):1725-30.
5. Afshar AR, Oh FS, Birnbaum AD, Namavari A, Riddle JM, Djalilian AR. Assessing ophthalmoscopy skills. Ophthalmology. 2010;117(9):1863.
6. Gupta DK, Khandker N, Stacy K, Tatsuoka CM, Preston DC. Utility of combining a simulation-based method with a lecture-based method for fundoscopy training in neurology residency. JAMA Neurol. 2017;74(10):1223-7.
