# Supplementary material for: Effect of the iExaminer Teaching Method on Fundus Examination Skills: A Randomized Clinical Trial
Source: JAMA Netw Open. 2019 Sep 20;2(9):e1911891. doi: 10.1001/jamanetworkopen.2019.11891 (PMC6755710; doi:10.1001/jamanetworkopen.2019.11891)
Supplement: Supplement 2. — eMethods. Study Flow Diagram [file jamanetwopen-2-e1911891-s002.pdf]

Supplementary Online Content

Shikino K, Suzuki S, Hirota Y, Kikukawa M, Ikusaka M. Effect of the iExaminer teaching method on fundus examination skills: a randomized clinical trial. *JAMA Netw Open*. 2019;2(9):e1911891. doi:10.1001/jamanetworkopen.2019.11891

eMethods. Study Flow Diagram

This supplementary material has been provided by the authors to give readers additional information about their work.

© 2019 Shikino K et al. *JAMA Network Open.*

eMethods. Study Flow Diagram

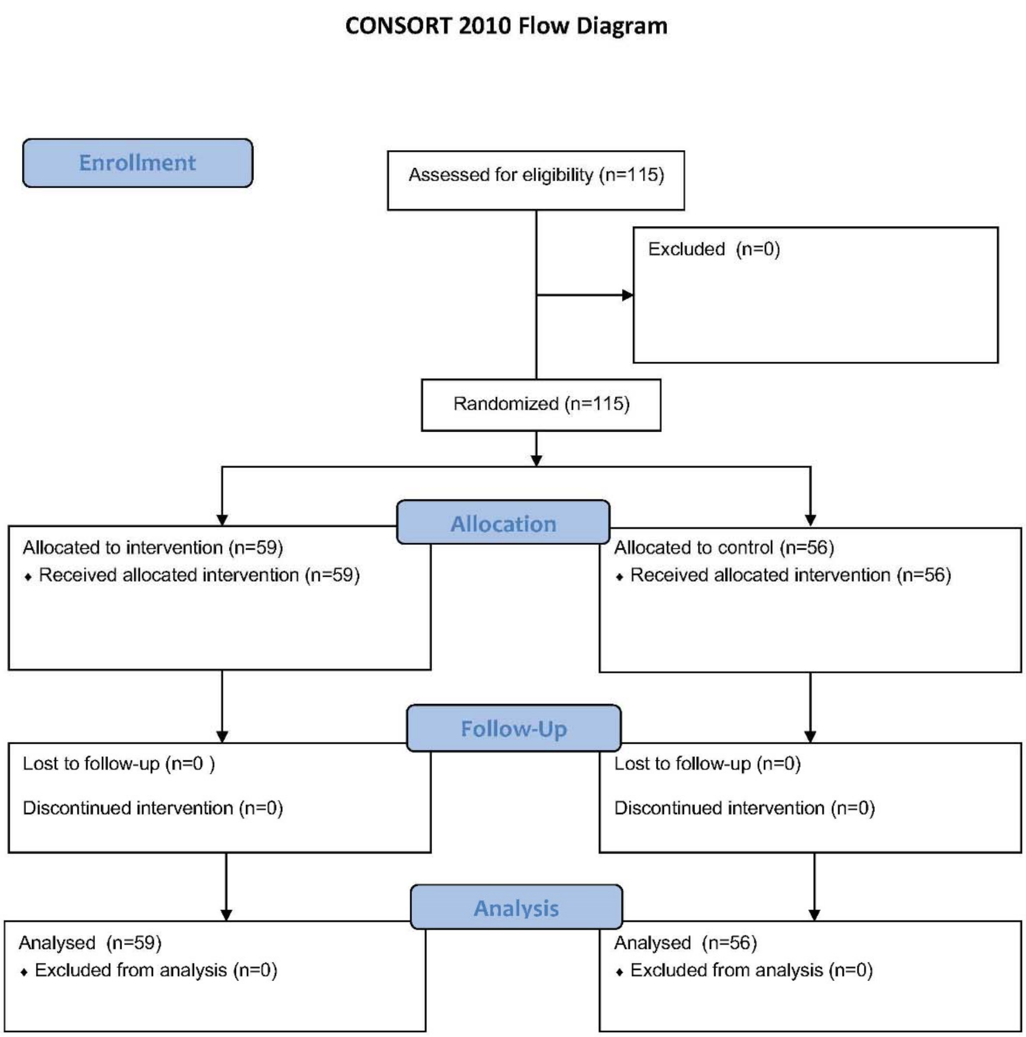

© 2019 Shikino K et al. *JAMA Network Open.*
