# Supplementary material for: Effect of the iExaminer Teaching Method on Fundus Examination Skills: A Randomized Clinical Trial
Source: JAMA Netw Open. 2019 Sep 20;2(9):e1911891. doi: 10.1001/jamanetworkopen.2019.11891 (PMC6755710; doi:10.1001/jamanetworkopen.2019.11891)
Supplement: Supplement 3. — Data Sharing Statement [file jamanetwopen-2-e1911891-s003.pdf]

Data Sharing Statement

Shikino. Effect of the iExaminer Teaching Method on Fundus Examination Skills. *JAMA Netw Open*. Published September 20, 2019.
10.1001/jamanetworkopen.2019.11891

Data
Data available: No
